# An extra hour wasted? Bar closing hours and traffic accidents in Norway

**DOI:** 10.1002/hec.4550

**Published:** 2022-06-01

**Authors:** Colin Green, Lana Krehic

**Affiliations:** ^1^ Department of Economics Norwegian University of Science and Technology Trondheim Norway; ^2^ NTNU Social Research Trondheim Norway

**Keywords:** alcohol policy, closing hours, traffic accidents

## Abstract

Driving under the influence of alcohol is a major cause of fatalities worldwide. There have been a range of legislative and policy interventions aiming to address this. Bar closing hours is one policy with clear implications for drink driving. Existing evidence, largely drawn from one‐off policy changes in urban settings, reports mixed evidence that is difficult to generalize. We return to this issue using a setting, Norway, that is advantageous due to large temporal and regional variation in closing times, frequent changes, and a lack of confounding policy changes. We demonstrate an average zero effect of closing hours on traffic accidents that masks large variations in effects: in terms of population density; accident severity; and direction of change in closing hours. Extensions in closing hours in populous municipalities decrease accidents, whereas the opposite is true for rural municipalities. Our findings suggest that estimates from single policy changes may be difficult to generalize, while demonstrating that closing hours can generate large effects on traffic accidents.

## 1 INTRODUCTION

Driving under the influence of alcohol remains a leading cause of fatalities and serious injuries worldwide. According to the World Health Organization (2007), approximately 20% of fatally injured drivers in high‐income countries have a blood alcohol concentration above the legal limit. Furthermore, over half of fatal accidents in the US happen at night‐time, where 54% of accidents are alcohol‐related (Forbes, 2009). Compared to the US, Norway has a lower prevalence of drink‐driving. Nevertheless, 25% of drivers involved in fatal car accidents had a blood alcohol concentration above the Norwegian legal limit of 0.02%. Moreover, in a survey of young drivers in Norway, nearly 10% stated that they have driven after consuming alcohol during the last 12 months (Norwegian Institute of Public Health, 2018). Consequently, policies aiming to prevent drink‐driving and reduce the risk of accidents remain a central part of the debate on appropriate alcohol policies.

The forefront of attempts to reduce these fatalities have been interventions aimed at reducing drink‐driving, mainly through lowering the blood alcohol level permissible while driving and attaching large penalties to breaches of these restrictions. Along these lines, the past decades have seen a general move to more stringent drink‐driving laws, and these have been shown to be effective in reducing traffic accidents and injuries. At the same time, a broader range of legislation on alcohol consumption has the potential to substantially impact upon drink‐driving and associated societal harms. One such area is on‐premises licensing laws. Where and when individuals can purchase and consume alcohol, such as at a bar, restaurant, or hotel, has natural links with drink‐driving. Moreover, while drink‐driving laws have become stricter, there has been a move toward more liberal closing hours of licensed premises in many jurisdictions. As an example, several US states (California, Michigan, Ohio, and Wisconsin) have bills currently in the legislative process aimed at extending closing hours.

In practice, the relationship between closing hours, drink‐driving, and traffic injuries is complicated by several factors. Longer opening hours are linked to greater alcohol consumption with the associated heightened drink‐driving risk. Yet, early closing times are often thought to result in so‐called drinking ’against the clock’. This has the potential to lead to increased inebriation at closing time and heightened risk of traffic accidents. More generally, unified and early closing hours increase risks related to multiple drinkers driving at the same time (Levitt & Porter, 2001). Stringent closing hours may also make multiple‐vehicle accidents more likely due to the higher underlying traffic flows present earlier at night. Yet, later hours may limit alternative modes of transport (public transport) or make it more expensive (taxis). Together this means that the direction of effect between closing hours and traffic accidents is unclear, and the existing evidence reflects this point.

A body of research has developed that seeks to provide causal estimates of closing hours on traffic accidents and fatalities. These papers typically have examined individual events of licensing changes. For example, Vingilis et al. (2005) investigate the effect on road safety of an extension in on‐premises alcohol sales from 1 to 2a.m. in Ontario, Canada. They find no impact on traffic fatalities with positive blood alcohol concentration after the extension. In a second paper, Vingilis et al. (2006) study accidents in Windsor, Canada following a harmonization in closing hours with bordering, later‐closing, Detroit. This extension led to an increase in accidents in the Windsor region. Yet, accidents in Detroit were reduced, likely reflecting reductions in Canadian patrons crossing the border to take advantage of the longer on‐premises closing hours. Bouffard et al. (2007) examine the effect of extended closing hours in Minnesota on police stops for Driving Under the Influence (DUI). While they demonstrate a significant increase in the number of police stops for DUI following this legislative change, further analyses suggested that this increase may largely reflect the increased policing that accompanied the policy change. Green et al. (2014) explore the effects of a large liberalization in bar closing hours, from 11p.m. up to 5a.m. that occurred simultaneously across all of England and Wales.^1^ They demonstrate marked reductions in traffic accidents and injuries. At the same time, Biderman et al. (2010) examine the effect of a restriction of bar closing hours in the Sao Paulo Metropolitan Area and demonstrate a reduction in fatal traffic accidents. Together, this highlights the mixed evidence of the effect of closing hours on traffic accidents. Related research shows that on‐premises availability increases accidents in settings likely to encourage driving to and from venues. For instance, Cotti and Walker (2010) demonstrates that casino openings lead to marked increases in drink related traffic fatalities within US counties. Both Cotti and Walker (2010) and Burton (2020) show that non‐uniform smoking bans increase drink‐driving traffic accidents, likely through increased distance individuals drive to consume alcohol in combination with smoking.

We return to this issue focusing on Norway. This provides an advantageous focal point for a variety of reasons. First, on‐premises closing hours are set at a highly disaggregated municipal level and vary considerably over time. This provides substantial variation in closing hours with changes in different directions and at different margins. Second, many other potential policy changes likely to confound estimates of the effect of on‐premises hour do not vary at the municipal level. This contrasts with many other settings, where drink‐driving penalties, drink‐driving limits, and off‐premises alcohol laws may be changed at the same time, or as a result of, on‐premises law changes. This makes it difficult to disentangle the effect of changes in closing hours from other changes in alcohol related policies. In Norway, these types of policies are set nationally and simply do not vary within our period of analysis. Third, the organization of policing, including the monitoring of drink‐driving, occurs at a higher regional level than the municipal level that bar closing hours are chosen at.^2^ This makes it less likely that our results reflect joint policing and bar closing hour decisions. Together, we argue that this provides a clean setting to isolate the effect of on‐premises closing hours on individual behavior. On top of this, there is marked regional variation in population density with commensurate differences in the density of off‐premises venues and availability of public transport. We utilize these differences to understand likely mechanisms generating our results. This is important when discussing the implications of our results for other jurisdictions.

Municipalities in Norway are free to choose closing hours within a broad nationally set limit of midnight to 3a.m. Moreover, local political candidates frequently include changes in bar hours as part of their political platforms. This leads to substantial variation both across municipality and time. Our main approach utilizes this within municipal across time variation to estimate the effect of different closing hours on a range of accident types and injury outcomes. We use panel data on closing hours covering 2009–2018 for 423 municipalities in Norway and combine it with detailed data on all reported night‐time traffic accidents. We demonstrate an average zero effect that hides marked variation across jurisdictions. Longer hours in highly populated urban areas are associated with pronounced lower accident and injury rates. In contrast, longer hours increase accidents in less populated areas. These results are robust to a range of likely confounding influences and remain in alternative data sources such as police DUI reports. Thus, later bar closing hours tend to reduce accidents due to a spreading of patron dispersal from bars and conditional on having public transport alternatives. In less populated municipalities, where public transport supply is low or even non‐existing, extended closing hours tend to exacerbate night‐time traffic accidents.

Our data and setting allow us to further explore several issues, particularly in terms of heterogeneous effects, that are largely missing from the existing literature. These range from asymmetric effects of bar hour extensions and restrictions; variations across population size of affected areas; and variations in effects on accident type and severity. As examples we show zero to little effect of restrictions on accidents, yet that liberalizations decrease accidents particularly in populous, urban settings. In addition, bar closing hours appear to have especially large effects on the likelihood of multiple‐car accidents. More generally, we demonstrate how closing hours can have dramatically different effects even within one national setting. This may provide an indication as to why existing research shows such mixed results of bar closing hours on traffic accidents.

In what follows, we describe the institutional framework and outline our data. This is followed by a description of our empirical methodology, our main results, robustness checks and examination of heterogeneity in treatment effects. We then provide a conclusion.

## 2 INSTITUTIONAL FRAMEWORK AND THE DATA

According to Norwegian law on‐premises alcohol sales are permitted between 8 and 3a.m. for beverages with an alcohol content up to 22%, such as beer and wine. Spirits containing between 23 and 60% alcohol can be served between 1p.m. and 3a.m.^3^ Municipalities are free to decide serving regulations within these hours, and serving hours can differ between beer and wine, and hard liquor, in the same municipality. Serving hours can also differ between weekdays and weekends. Our main approach, unless stated otherwise, is to use weekend closing hours for hard liquor. We stress, however, that our results are unchanged if we instead use the beer and wine serving hours.

Unlike on‐premises laws, Norway has a national alcohol policy with respect to off‐premises alcohol sales. Beer (up to 4.7% alcohol content) cannot be purchased off‐premises after 8p.m. on weekdays, 6p.m. on Saturdays and not at all on Sundays. Other stronger alcohol can only be purchased from the government run monopoly which exists in few locations (e.g., Trondheim with 200,000 inhabitants has eight of these shops) and with very limited opening hours (weekdays until 6p.m., 10am‐3pm on Saturdays and closed on Sundays). These off‐premises laws remain unchanged across our period of analysis.

Changes to on‐premises closing hours in Norwegian municipalities are frequent and are often subject to political debate and media attention (Rossow & Baklien, 2014). These changes often, but not exclusively, take place shortly after a new council has been elected, suggesting that alcohol policy is part of many local election campaigns. In general, the reasons behind the decision to alter closing hours are multi‐factorial, and the public debate is often divided between public health and industry interests. Proponents of liberal closing hours argue that it contributes to the liveliness of cities and increases revenue for hotels, restaurants, and bars. Opponents argue that earlier closing hours decrease street violence and a range of other social externalities including drink‐driving. Support for both arguments can be found in the small, associative, literature that exists for Norway. For instance, Melberg and Schøyen (2012) find that a reduction in alcohol serving time by one hour is associated with reduced revenue of between 9% and 12%. On the other hand, Rossow and Norström (2012) find that a one‐hour extension in closing hours is associated with an increase in assaults of 13%.

Data on closing hours comes from local council responses to the Alcohol Act survey, conducted by the Norwegian Institute of Public Health. The questionnaire is sent to all Norwegian municipalities every year, with a very high response rate of between 93 and 99%. We use the information on closing hours from 2009, the earliest year for which information on municipal closing hour is available, to 2018. During this period there were 434 individual municipal changes in on‐premises serving hours. This is equivalent to each municipality changing their closing hours, on average, one time over the 10‐year period.

**FIGURE 1:**
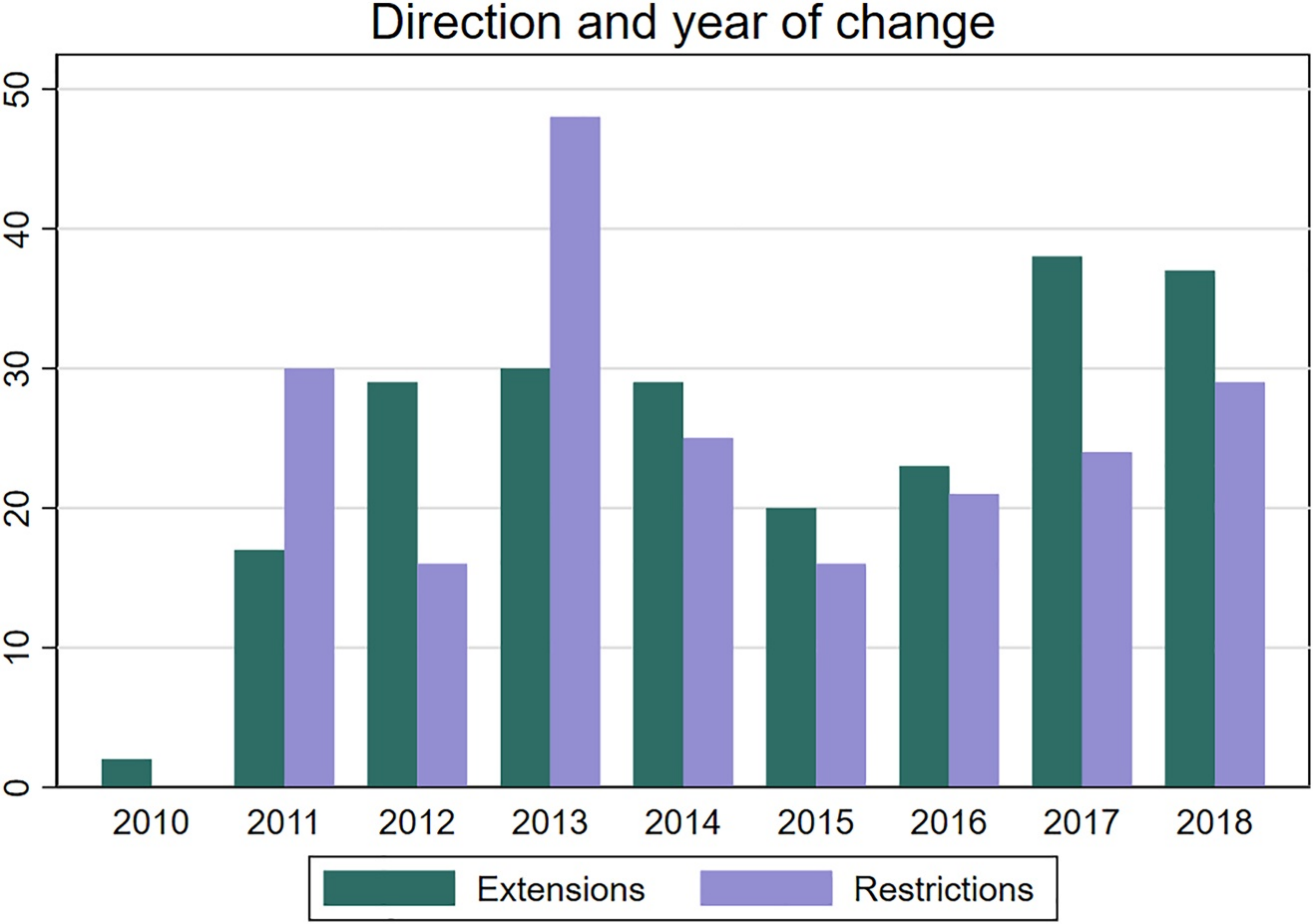
An overview of the number of changes in different directions by year

Because changes in closing hours are at the discretion of local municipal councils across Norway, we might expect both the timing and direction of changes to have a relatively uniform distribution across our sample period. Figure 1 shows the number of changes in closing hours per year whilst also illustrating the direction of the change. Overall, there seems to be no clear trend in changes over time, nor is there evidence of a preference for changing closing hours in one particular direction. On average, there are 50 changes each year. Two years, however, stand out in terms of number of changes, namely 2010 and 2013. It is difficult to know if this is simply chance or reflect other factors. In all estimates we include year dummies to control for potential unobservable national factors that might affect the decision to change closing hours.

**FIGURE 2:**
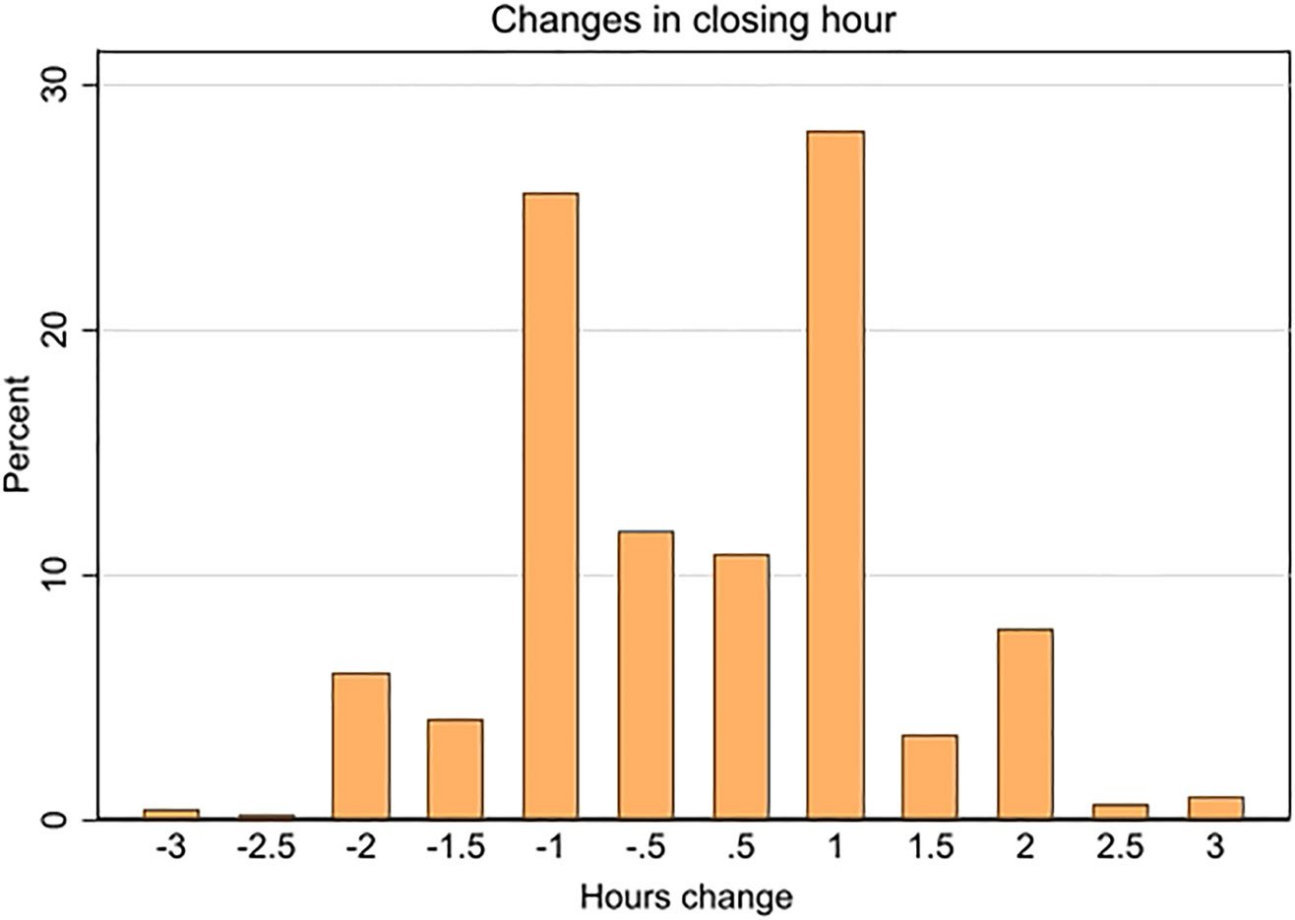
A detailed overview of the direction of change in closing hours

Figure 2 provides more information in terms of both the magnitude and the direction of change in closing hours. The changes are quite evenly split between reductions and extensions.

Around 55% of changes involve increases or decreases of on‐premises sales by one hour, 22% are 30‐min changes, and there is a non‐trivial number of changes of more than one hour in terms of both extensions and restrictions. This allows us to examine the effects of a range of changes, for example, in different directions, on the effect of closing hours on traffic accidents.

**FIGURE 3:**
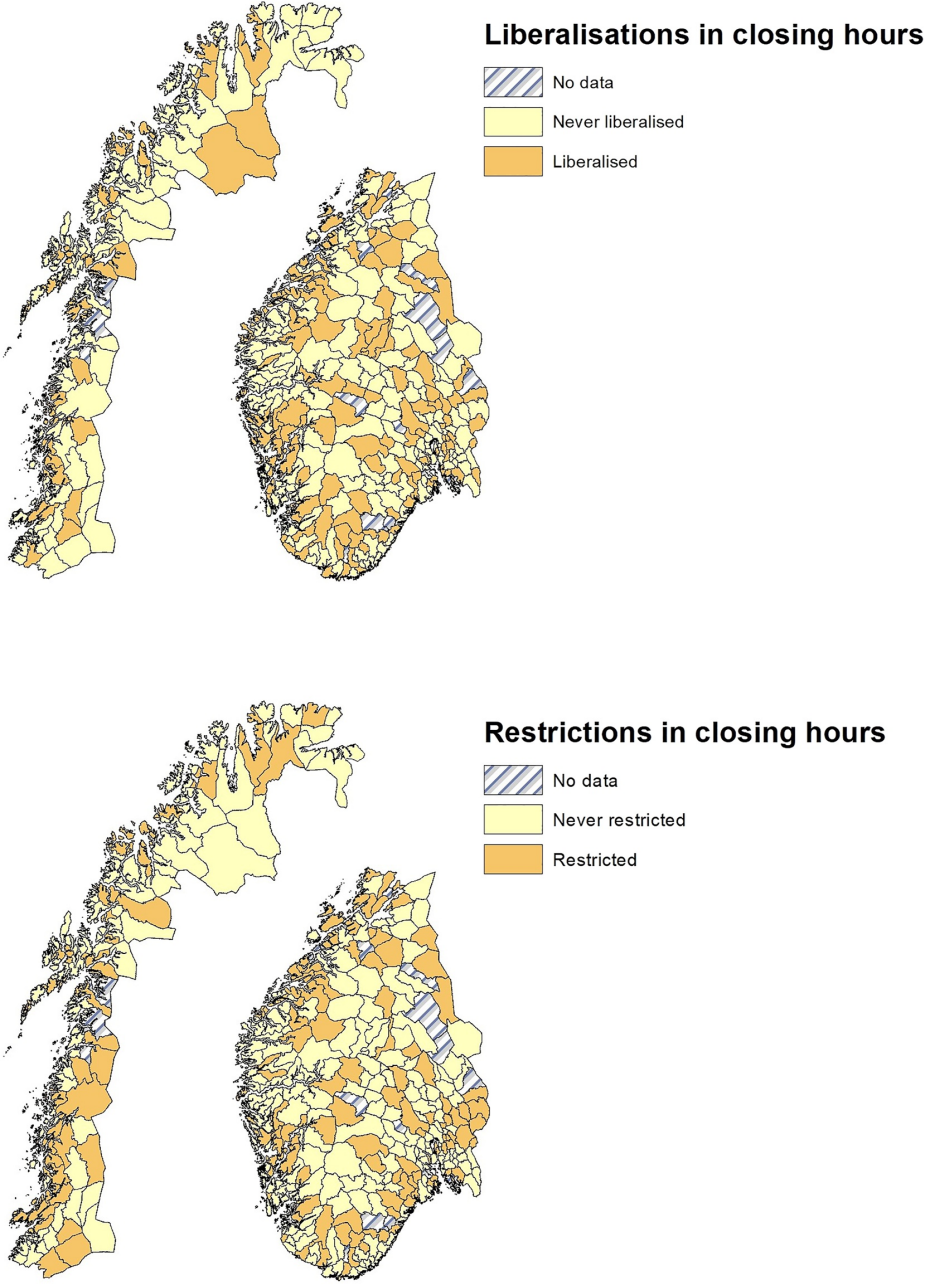
Geographical distribution of municipalities that ever liberalized and restricted their closing hours

Figure 3 provides geographic information on these changes where for presentation purposes we separate northern and southern Norway. The upper panel displays municipalities that at some point during the sample period extended on‐premises serving hours.^4^ In the lower panel we plot municipalities that have restricted their closing hours. Two points can be made from these maps. First, municipalities that liberalized or restricted hours are spread across the country. This reduces concerns that changes are geographically clustered in some manner, for instance around major cities, in areas with strong religious preferences, or in areas with a strong brewing industry. Second, and although more difficult to see graphically, a non‐trivial number of municipalities have both extended and restricted closing hours during the period (158 municipalities).

Our road accident data comes from The Norwegian Public Roads Administration (NPRA) and contain all motor vehicle accidents reported to the police from 2009 to 2018 for all 423 municipalities. We have information on the date and time of accident, severity, road speed limit and accident location. This data allows us to match accident location to the corresponding municipality's closing hours. We match the accidents and closing hour data to population level data from Statistics Norway, and data on the number of individuals between the age of 18 and 25 to construct a variable of the share of young adults in the municipality. This reflects the fact that young drivers are both much more likely to be involved in a traffic accident than other sober drivers and may be more affected by changes in on‐premises closing hours. Notice that, even though the accidents data is detailed with respect to month and day of an accident, the closing hours survey does not collect information on the date of a potential closing hour change, only that the hours have changed from 1 year to another. This leads us to conduct our analysis at a year level.

Systematic differences between those municipalities that did and did not change closing hours can bias estimates. Table 1 displays summary statistics of the variables included in our analysis, divided by whether municipalities have changed their closing hours. Approximately half of the municipalities have not altered closing hours during the sample period. No change municipalities have slightly more accidents and have somewhat more liberal closing hours. It is worth noting that population size, is on average, larger for municipalities that do not change closing hours. Controlling for population and including municipality fixed effects should account for related confounding factors that might bias the estimates of the effects of closing hour.

**TABLE 1:** Descriptive statistics by closing hours status (2009–2018)

	(1)	(2)	(3)
	All	Unchanged	Changed
Municipalities	423	204	219
Accidents	0.82	0.96	0.68
	(2.60)	(3.37)	(1.58)
Closing hour (beer/wine)	2.02	2.05	1.98
	(0.60)	(0.57)	(0.63)
Closing hour (spirits)	1.73	1.78	1.67
	(0.82)	(0.79)	(0.86)
Population	11,943	14,553	9508
	(36,561)	(48,031)	(20,495)
Young adults	1247	1540	972
	(4056)	(5287)	(2362)

*Note*: The variable *Accidents* is the number of traffic accidents occurring weekends between 10p.m. and 5a.m. in a municipality over the course of 1 year. Standard deviations in parentheses.

Population‐size variation within Norway is worthy of further discussion as Norwegian municipalities vary greatly in size and population. For example, the least populated municipality has 200 inhabitants, whereas 658,000 people live in the most populated municipality. In fact, the smallest half of Norwegian municipalities account for approximately 10% of the entire population of Norway.

**FIGURE 4:**
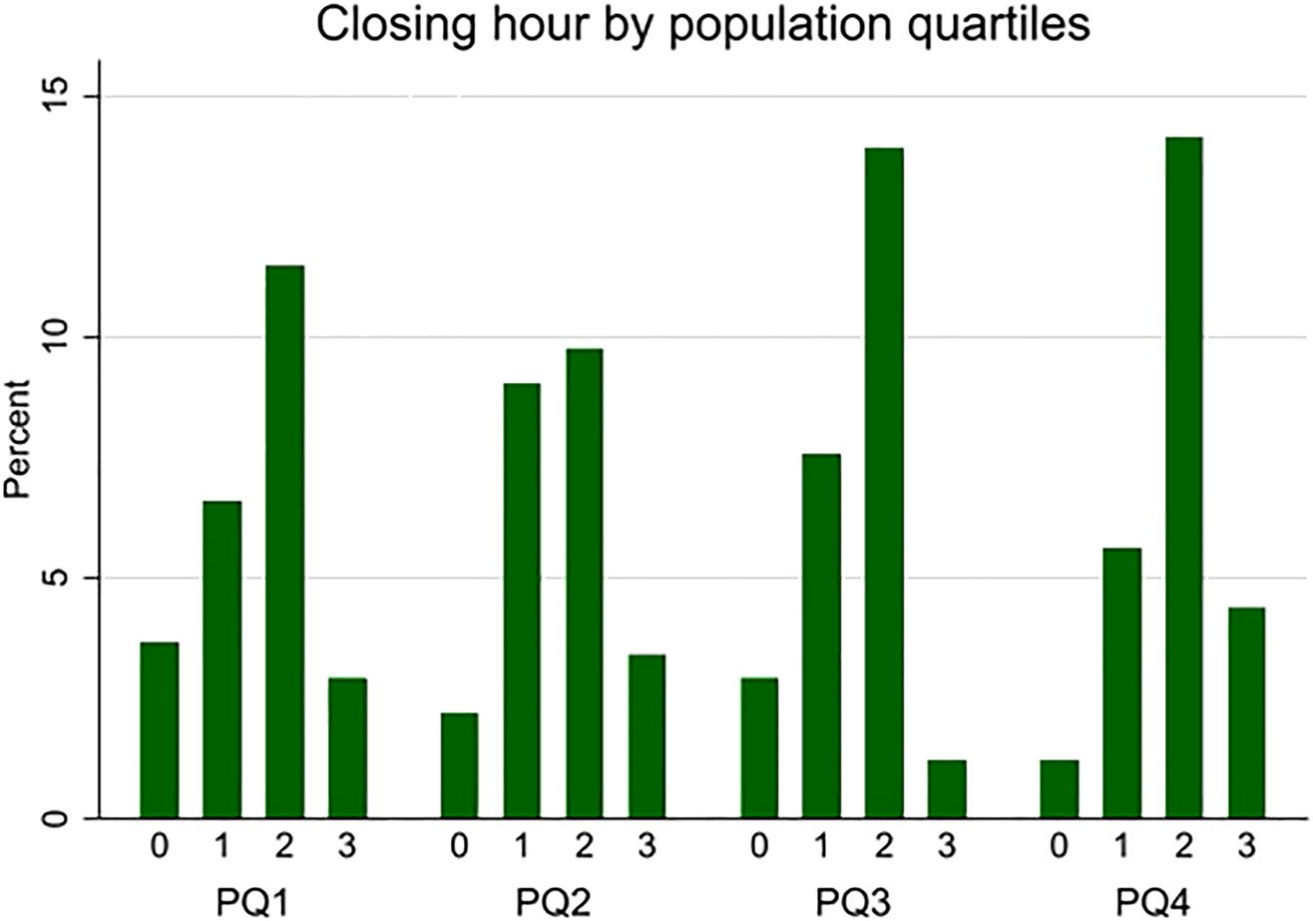
An overview of closing hours according to population size quartiles

Figure 4 presents an overview of closing hours according to different municipal population quartiles in the middle year of the sample, 2014.^5^ There is no evidence that, for example, only the most populous municipalities close at the latest permissible hour (3a.m.). Although municipalities belonging to different population quartiles have similar variation in closing hours, there are other differences related to population size that are germane to our analysis. For instance, in small to mid‐sized municipalities, there are no buses, trains or trams to take bar patrons home after the bar closes. With approximately 20,000 inhabitants, Grimstad is to our knowledge the smallest town (and municipality) that provides night‐time public transport. Consequently, taxis may in most other small places be the only viable public transport alternative.^6^ If there is a night‐time taxi service available, which is not always the case, there is likely to be queuing around closing time. Moreover, people often live scattered within the least populous municipalities, meaning that taxi costs can be prohibitive especially when coupled with average taxi driver wages in Norway. For example, a weekend night‐time taxi ride of 6.3 km (4 miles) with a duration of 15 min is estimated to cost 400 Norwegian Kroner (NOK) (approximately 45.7 USD).

A 30‐min ride that covers approximately 20 km (12.4 miles) costs 1145 NOK (131 USD). In summary, night‐time public transport is often not a realistic alternative in sparsely populated municipalities. This might translate to heterogeneous effects of changes in closing hour on night‐time traffic accidents between rural and urban areas, which will be important to account for in our analysis.

## 3 EMPIRICAL APPROACH

Our main approach is to estimate variants of:

(1) $$Acc_{it}=\beta_{0}+\beta_{1}Closing{Hour}_{it}+\beta_{2}Closing{Hour}_{it}\;•\;Population_{it}+\gamma X_{it}+\alpha_{i}+\delta_{t}+\varepsilon_{it}$$

where *Acc*_*it*_ is the number of weekend accidents happening between 10p.m. and 5a.m. in municipality *i* in year *t*. *ClosingHour* is the maximum allowed on‐premises alcohol serving hour during weekends in municipality *i* in year *t*, ranging from midnight to 3a.m. Vector *X* includes the population level and the number of young adults (aged 18–25) in municipality *i* in year *t. α*_*i*_ and *δ*_*t*_ captures municipal and year fixed effects, respectively, and *ε*_*it*_ is random idiosyncratic error. Hence, our estimates of interest, *β*_1_ and *β*_2_, are identified by within municipality variation in closing hours holding constant nationwide annual patterns in accidents.

We suspect that any effect of bar closing hours is heavily dependent on municipal population size for the reasons we described earlier. These include the fact that more populous municipalities are likely to have higher concentrations of bars, but also have substantially greater public transport availability at closing times attenuating any effect on drink driving. In rural, less populated, areas, typically there will be no public transport provision and distances from bars to home may be substantially greater, both increasing the risk of accident and the cost of taxis (if available). In practice, there does not exist an obvious way to model and capture these differences. Our initial approach is to include an interaction term of bar closing hours and population in our main model, with the aim of examining if there is any evidence of heterogeneous effects of closing hours by population. Subsequently, we examine the role of factors such as access to public transport at bar closing times.

Our choice of municipal fixed effects approach is motivated by a range of concerns regarding cross municipal variation in time‐invariant factors influencing alcohol consumption, bar closing hours, and underlying risk of traffic accidents. For instance, there is variation across Norway in the strength of religious attitudes, which simultaneously influences drinking culture, closing hours, and night‐time activities. Some municipalities are in locations where the difficulty of driving, and the risk of accident, is higher. This leads to a concern that OLS estimation will overstate the effect of bar closing hours on traffic accidents. Alternatively, some municipalities in rural areas might have a strong drinking culture with liberal bar closing hours, but also a higher accident prevalence because of lesser provision of night‐time public transport services. Again, this may lead to OLS estimates being upwardly biased.

A further concern, not mitigated by the fixed effects approach, is the potential for time varying factors correlated with both closing hours and traffic accidents. For example, nationwide shocks, caused by government awareness campaigns or increased taxation on alcohol, could reduce closing hours and the number of traffic accidents in several municipalities within the same year. We include year fixed effects to capture such influences that are common for all municipalities. Municipal specific time varying factors are more difficult to address, and it is unclear in what direction these may bias our results. For example, restrictions of hours and the election of municipal governments that favor restrictions may gain more traction in instances where there have been increases in underlying problems related to alcohol consumption in the local area. At the same time, increases in local economic activity may increase the number of licensed venues, and increase pressure on municipal governments to extend hours. We adopt several approaches aimed at assessing the sensitivity of our main estimates to these types of factors. These range from including municipal specific time trends to estimating disaggregated models for liberalizations and restrictions. While these tests do not directly address time varying unobservables, they provide some gauge of the sensitivity of our main estimates.

## 4 RESULTS

**FIGURE 5:**
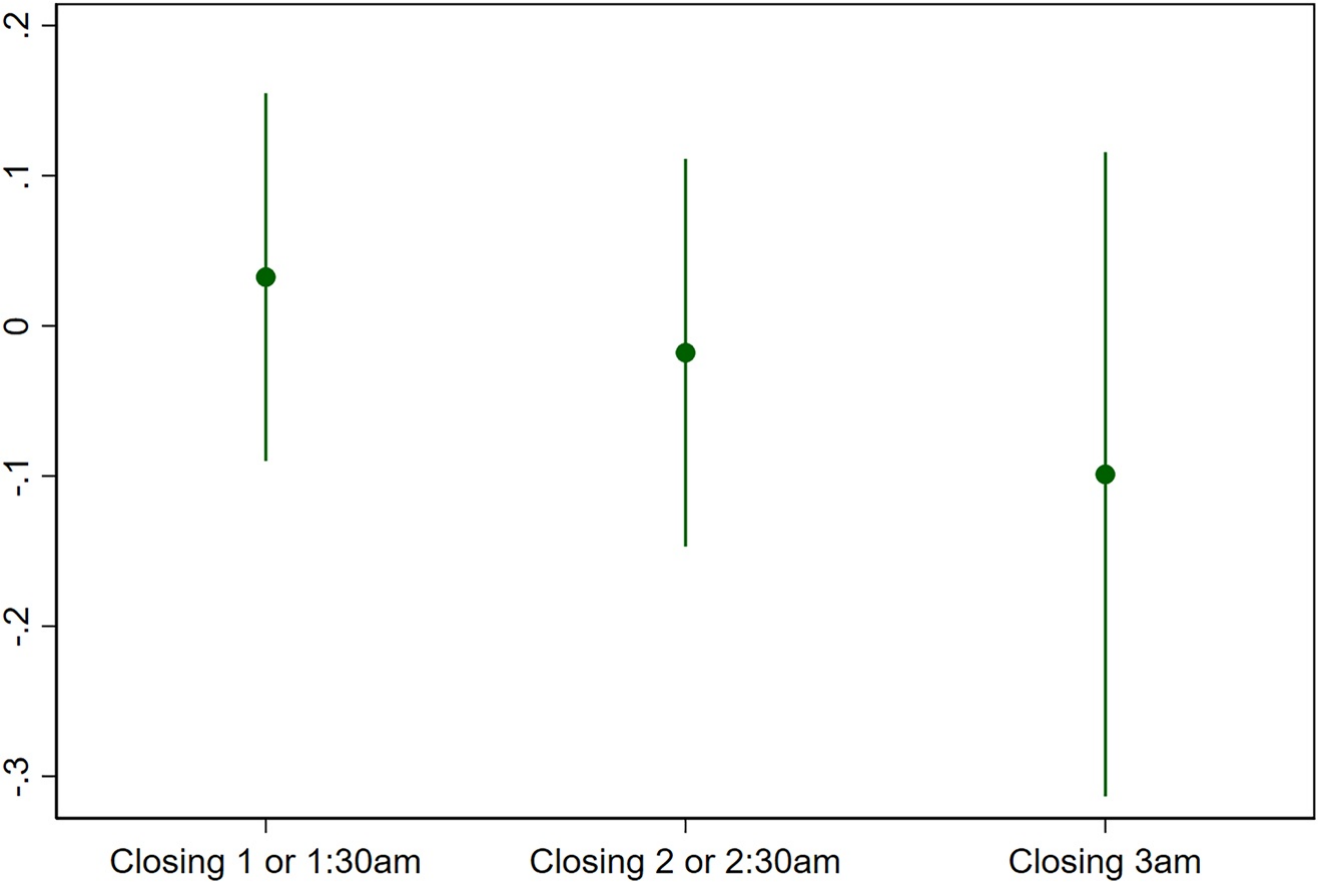
Estimated impact of different closing hours on traffic accidents

Column (1) in Table 2 provides initial estimates of the relationship between closing hours and night‐time traffic accidents. The standard errors are clustered at the municipal level.^7^ The result suggests a negative, however small, and not statistically significant, relationship between longer opening hours and accidents. This estimate shows the linear effect of increasing closing hours by one hour and may hide marked non‐linearities across actual closing time. To examine this, we additionally estimated an analogue of (1) where we replaced closing hours with a series of dummy variables to indicate closing hour, where midnight is the omitted category. These estimates are plotted in Figure 5, which depicts a coefficient plot with confidence intervals at the 95% level. They suggest no difference between closing times of midnight, 1a.m. or 2a.m., but some suggestion of lower accidents at the latest closing time. However, none of these coefficients are statistically significant at standard levels, and the estimate for 3a.m. closing is particularly imprecise.

**TABLE 2:** The influence of bar closing hours on traffic accidents between Friday and Sunday (2009–2018)

	Hard liquor			Beer and wine
	(1)	(2)	(3)	(4)
Closing hour	−0.004 (0.033)	0.382*** (0.071)	0.137*** (0.046)	0.128*** (0.043)
Closing hour × population		−0.536*** (0.091)	−0.187*** (0.064)	−0.236*** (0.052)
Population (/10,000)			−3.496*** (0.510)	−3.397*** (0.439)
Number of young adults (/10,000)			13.195* (7.150)	13.552** (6.872)
Constant	1.123*** (0.090)	1.795*** (0.177)	3.890*** (0.361)	3.878*** (0.393)
Year dummies	Yes	Yes	Yes	Yes
R2	0.019	0.116	0.167	0.169
Observations	4039	4039	4039	4039
Municipalities	423	423	423	423

*Note*: The dependent variable is the number of traffic accidents occurring weekends between 10p.m. and 5a.m. ***, **, * indicate statistical significance at 1%, 5% and 10%, respectively. All models include municipal fixed effects.

As discussed earlier, it is likely that any relationship between closing hours and accidents may vary according to population levels and the number of young adults in each municipality. As a first step to investigating this, we allow the effect of closing hours to vary by the population size of the municipality. In column (2), we present the results where we include an interaction between municipal population and bar closing hours. This dramatically changes the estimate of interest. The initial effect of liberalizing bar closing hours is positive and statistically significant at the one percent level, implying an increase in 0.4 accidents when extending bar closing hours by 1 hour. The interaction term between bar closing hours and population is negative, statistically significant, and sizable. Thus, the positive effect of bar closing hours decreases as population size increases, with the effect on average being zero in municipalities with approximately 7100 inhabitants and turning negative in more populous municipalities. This provides an initial suggestion of marked differences in the effect of closing hours by municipal setting. For example, for the most populous municipality in Norway (Oslo), the results suggest approximately 37 accidents less per year after liberalization, or three night‐time accidents less per month.^8^ At the same time, there are 230 municipalities in Norway with less than 5000 inhabitants. Extending closing hours would increase accidents by 30% for these municipalities, adding up to 24 accidents for all municipalities. We next add population size and the number of young adults to the model in our municipal fixed effects strategy. These seek to capture potential confounding influences of demographic change on the number of night‐time traffic accidents. The result is presented in column (3), which is equivalent to Equation (1). The magnitude of both closing hours and the interaction term is reduced, but both direction and statistical significance are unaltered.^9^ This model is our main specification.

Municipalities are allowed to differentiate between serving hours for spirits, and for beer and wine. For example, beverages with lower alcohol levels can be sold later than hard liquor.^10^ For simplicity we have used hard liquor closing hours up to this point. In column (4), we present estimates where we use the applicable serving hours for beverages with a lower alcohol content. The key estimates of interest are essentially unchanged by this. As a result, we focus solely on the hard liquor hours from this point on but stress that all following estimates are essentially unchanged if we use these alternative hours.

## 5 ROBUSTNESS

### 5.1 Alternative specifications

We next test the sensitivity of the baseline results by estimating alternative specifications of the main model. First, we replace the linear interaction term of closing hour and population with discrete groups of population tertiles. This provides a useful test of robustness because the linear dose‐response interaction term can, for instance, mask large effects in the upper tail of the population distribution or other non‐linearities in the impact of population size.

**FIGURE 6:**
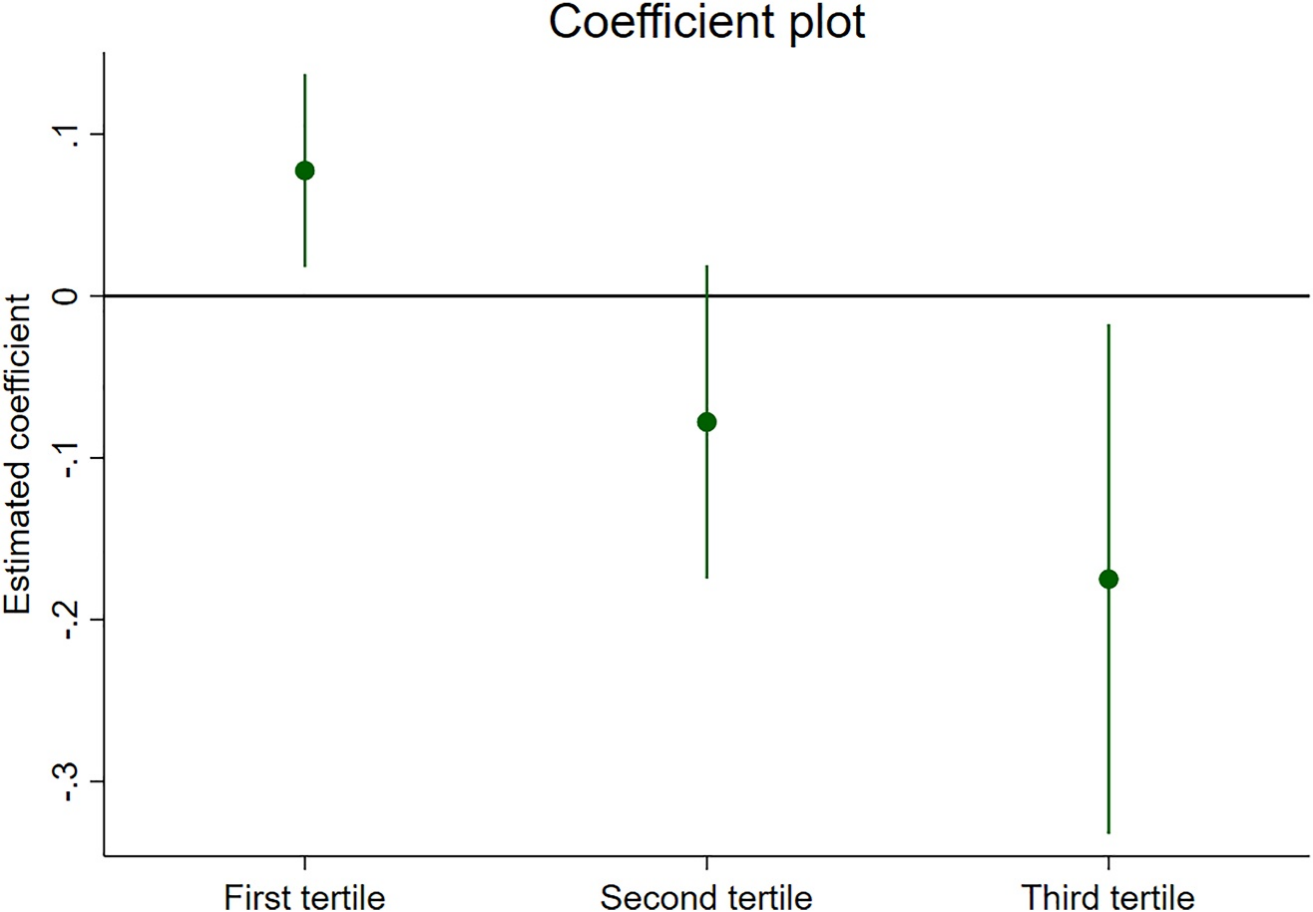
Coefficient plot of an alternative estimation approach using discrete population percentile interactions

The result, presented in Figure 6, largely fits with the earlier estimates.^11^ Less populated municipalities, with a mean of 1600 inhabitants, experience an increase in accidents when closing hours are extended. The opposite is true for the largest municipalities, that have an average of 30,000 inhabitants. Moreover, the estimate for the largest municipalities is larger in magnitude compared to the smaller municipalities. This suggests that the effect is not only heterogeneous but asymmetric according to municipal size. The coefficient for the municipalities belonging to the second tertile, with an average population of 5000, suggests a negative effect that just fails to be statistically significant at the five percent level. In general, these results offer some evidence that our simple interaction approach provides a reasonable approximation of the effect of population size in influencing the impact of closing hours on accidents. Nonetheless, we further explore effect heterogeneity by municipality characteristics in Section 6.

Table 3 provides variants of our main estimates motivated by a range of concerns of specification choice. In column (1) we change all variables, except for closing hours, to be expressed in natural logarithms.^12^ The result supports the findings from the main model, suggesting that an increase in closing hours increases the number of traffic accidents, but that the magnitude of this effect changes with population size with a turning point that is similar to that for the main estimates. Next, we re‐estimate our main model using population levels as weights.^13^ Comparing unweighted and weighted regressions is a useful diagnostic for model misspecification, as suggested by Solon et al. (2015). The coefficients of interest in column (2) increase somewhat compared to our main estimates, but the sign and significance of the coefficients persists.

**TABLE 3:** Alternative specifications of the effect of closing hours on traffic accidents

	Logs	Weighted	Daytime accidents	Municipal trends	Fixed population in 2009
	(1)	(2)	(3)	(4)	(5)
Closing hour	1.92** (0.889)	0.722*** (0.186)	0.157*** (0.044)	0.101** (0.040)	0.162*** (0.050)
Closing hour × population	−0.225** (0.110)	−0.469*** (0.107)	−0.188*** (0.065)	−0.178*** (0.053)	−0.231*** (0.075)
Weighted	No	Yes	No	No	No
Municipal trends	No	No	No	Yes	No
R_2_	0.029	0.424	0.287	0.324	0.168
Observations	4039	4039	4032	4039	4039
Municipalities	423	423	423	423	423

*Note*: All regressions are estimated with municipal fixed effects and include controls for population, number of young adults and year effects. ***, **, * indicate statistical significance at 1%, 5% and 10%, respectively.

The general risk of accidents could vary across municipalities over time, and this could, in turn, influence the number of night‐time traffic accidents. As a proxy for the accident risk in each municipality, we calculated the daytime accident rate (between 8 a.m. and 5 p.m.) during weekdays in each municipality and add this as a control to our main model. The result is shown in column (3). The variables of interest change negligibly, which suggests that there is no underlying time‐varying accident risk that confounds the main estimates. Next, there could exist unobserved trending factors that affect both closing hours and the number of traffic accidents, such as attitudes toward alcohol or local economic factors. Failing to control for these may lead to biased and inconsistent estimates of the effect of closing hours on traffic accidents. To examine this, we estimate our main model including municipal time trends. The coefficients presented in column (4) are marginally smaller in magnitude compared to the main result, yet both estimates are quite precisely estimated, and the patterns of effects remain essentially the same. This makes it less likely that our results are driven by, for instance, policy responses to trends in drink driving within municipalities.

A further test is related to our interaction of closing hour and population. As both variables vary over time it may be that some of the effect of the interaction term derives from variation in population levels instead of changes in closing hour. We explore this by fixing population levels in the interaction term at the first year of observation, so that only closing hour changes over time. The results of this approach, displayed in column (5), replicates the coefficients in the main results both in terms of magnitude and statistical significance.

### 5.2 Threats to identification

This section provides further checks aimed at examining threats to our identification strategy. As pointed out earlier, alcohol and drink‐driving policies are set at the national level. This greatly reduces the risk of a correlation between municipal closing hours and other related policies. However, there is a concern that closing hours are changed as a response to changes in the number of traffic accidents. Policymakers can alter closing hours as a means of counteracting a trend of increasing night‐time traffic accidents. This represents a potential reverse causality that can lead to a bias in our main estimates.

We examine this in two ways. First, we perform a text analysis in a media archive database for newspaper articles written during our sample period. When searching for the word “closing hour”, we found 1048 newspaper articles that contained this word during our sample period in a national, regional, or local newspaper in Norway.^14^ Next, we explored in how many of these newspaper articles the words traffic accident(s) or drink‐driving occurred, which returned a total of nine articles. This suggests that the public debate regarding closing hours is rarely related to the number of traffic accidents.

Second, we perform an event study to examine whether there is any evidence of increases or decreases in the number of traffic accidents the years before a change in closing hours. To facilitate this, we focus only on those municipalities that changed their closing hours just one time during the sample period. This leaves us with 96 municipalities. We split the sample into municipalities that reduced (36) and extended (60) their closing hours, respectively. The rationale is that, if municipalities are reacting accident trends, we might expect municipalities that experience an increase in accidents to react differently than municipalities that are experiencing decreases. The control variables included in the event study are the same as in the main model.

**FIGURE 7:**
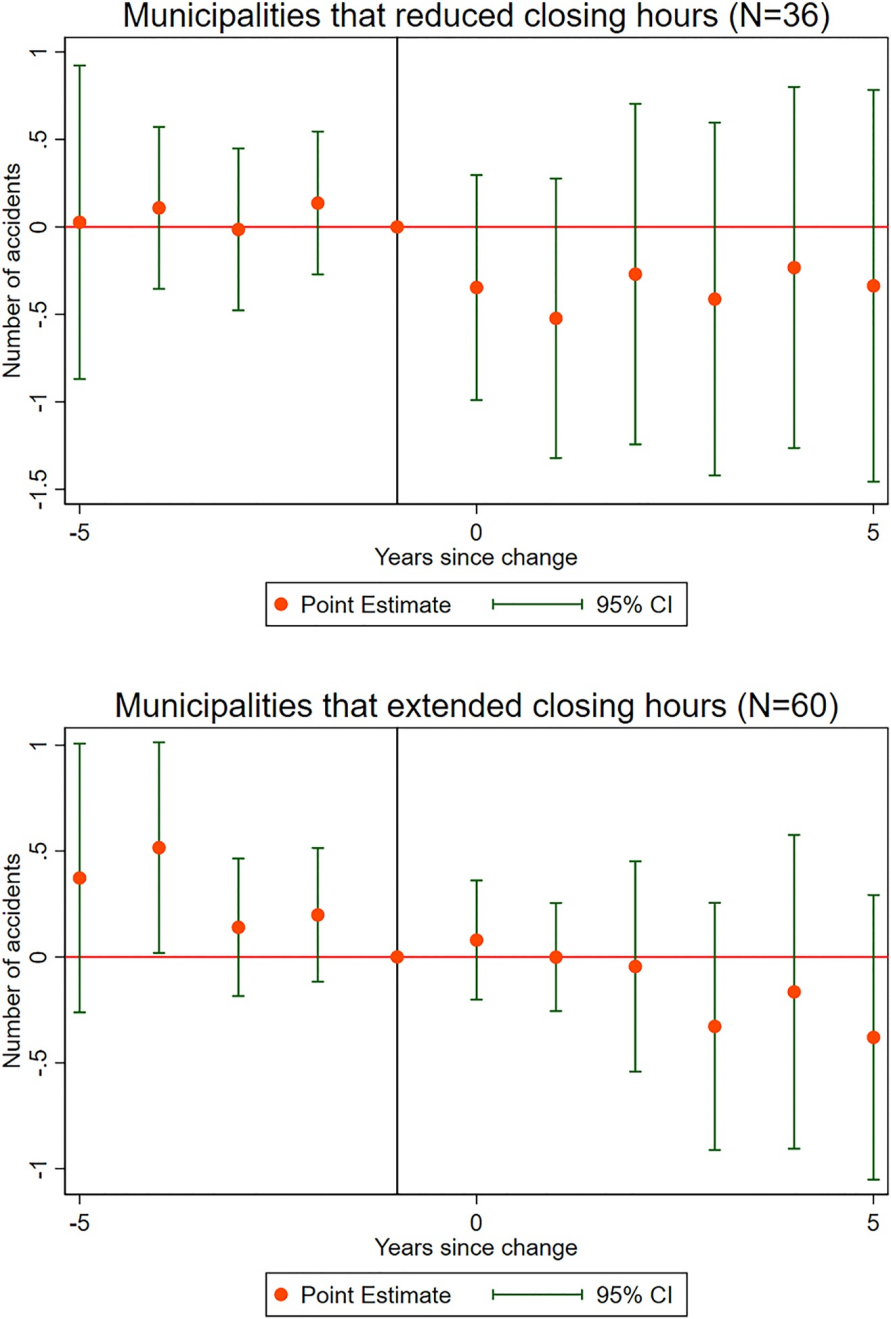
Event study of the number of accidents before and after the change in closing hours

The result of the two event studies is presented in Figure 7. The top graph shows the trend of night‐time traffic accidents 5 years prior and after closing hours are reduced, whereas the bottom graph shows accident trends related to extending closing hours. Neither graph exhibits any evidence of a clear trend preceding the change in closing hours. The analyses also suggest an average zero effect on accidents following changes in closing hour. However, the results should be interpreted with caution as the sample is relatively restricted, and the interaction term is not included in the model. Generally, the absence of a clear preceding trend suggests that politicians are not campaigning to change closing hours as a response to time variation in accidents.

One additional concern is that, because cities simply offer more night drinking opportunities, people living in rural areas may be more responsive to closing hours in nearby cities than in their own municipality. This could generate potential measurement error likely to bias our estimates of closing hours effects toward zero. At the same time, a change in closing hours that increases patronage from neighboring, rural municipalities might lead to an increase of accidents in the treated city. If this is the case, the estimate of the interaction term between closing hours and population could be biased upwards.

We explore this by dividing groups of Norwegian municipalities into 85 economic regions and assign the closing hours of the hub of the economic region to all municipalities in the region.^15^ The result of this exercise is presented in Table 4 where the second column reports population weighted results. In both models, the results are similar in spirit to the relevant estimates in Tables 2 and 3. Note that this should not be interpreted as meaning that local closing hours have no effect on accidents within smaller municipalities. Recall, for instance, that Figure 6 shows a clear positive effect of longer closing hours in less populated municipalities on accidents in those municipalities.

**TABLE 4:** Exploring possible spill over effects of closing hours

	Large city closing hours	
	(1)	(2)
	Accidents	Accidents
Closing hour	0.129** (0.059)	0.671*** (0.205)
Closing hour × population	−0.177** (0.064)	−0.458*** (0.103)
Weighted	No	Yes
R_2_	0.162	0.422
Observations	4039	4039
Municipalities	423	423

*Note*: The dependent variable is the number of traffic accidents occurring weekends between 10p.m. and 5a.m. Standard errors are clustered at the economic region level. ***, **, * indicate statistical significance at 1%, 5% and 10%, respectively.

A feature of the accidents data is that we do not observe whether the driver was impaired and whether their blood alcohol concentration level was above the legal limit.^16^ This means that the effect of closing hours on the number of accidents may reflect drinking behavior, or potentially other factors such as driver fatigue. To explore this further, we utilize police report data regarding motorists driving under the influence.^17^

There are two issues with this data. First, some charges are dropped if later testing (typically at a hospital) clears the driver of wrongdoing, yet these reports will remain in our data. Second, we cannot determine whether the driver was under the influence of alcohol or drugs, or both. Nonetheless, using this data provide additional information on the likely channels of the effect that we observe of closing hours on traffic accidents. We re‐estimate our main models with our dependent variable being the number of night‐time DUI reports. Column (1) in Table 5 presents these estimates. The estimated relationship between closing hours and DUI reports follows that for accidents. Longer hours lead to more DUI reports, but this effect decreases and becomes negative in more populous municipalities. The turning point of this relationship, approximately 6600 inhabitants, is very similar to that for our main models. This further suggests that there is substantial heterogeneity between the effect of closing hours in large urban municipalities, and smaller rural municipalities. At the extreme, these results imply a decrease of 61 police reports per year in Oslo when closing hours are extended by one hour.

**TABLE 5:** Examining the number of driving under the influence reports

	(1)	(2)
Closing hour	0.605*** (0.190)	1.944*** (0.462)
Closing hour × population	−0.915*** (0.263)	−1.322*** (0.232)
Weighted	No	Yes
R2	0.084	0.320
Observations	4036	4036
Municipalities	424	424

*Note*: All regressions are estimated with municipal fixed effects and include controls for population, number of young adults and year effects. ***, **, * indicate statistical significance at 1%, 5% and 10%, respectively.

A further concern is the potential for scale effects in the detection of DUI across municipalities of differing sizes. For example, a roadside breath‐test on 50 motorists in a less populated municipality, compared to a large one, will lead to an oversampling of drivers in the small municipality. We follow the approach suggested by Solon et al. (2015) and estimate a weighted regression with robust standard errors to obtain consistent coefficients. The results are presented in column (2). Once again, the direction of the estimates is the same as the results obtained in the main model. The coefficients are larger and statistically significant at the one percent level. These results suggest that driving under the influence is the likely transmission channel between closing hours and night‐time traffic accidents.

## 6 HETEROGENEITY

Our results to this point have shown that the effect of changes in bar closing hours depends on the population size in the municipality. Naturally, population may itself proxy for a range of factors that influence both drinking and driving behavior. One relevant factor is access to night‐time public transport, which varies clearly across municipalities. Moreover, it is likely that the night‐time public transport supply impacts our main results, as night bus provision has been shown to reduce traffic related injuries and fatalities (Lichtman‐Sadot, 2019).

In this analysis, we investigate whether the population size effect found in the main results reflects municipal differences in night‐time public transport provision. We start by creating two groups of municipalities. The first group consists of 10 municipalities that we know have a year‐round night‐time mass public transit supply, such as buses, trams, or subways. The other group constitutes the 25% least populated municipalities (less than 2000 inhabitants), where night‐time public transport is likely to be non‐existent. We estimate our main model on the two samples without the population interaction term. The results are presented in Table 6. The direction and the magnitude of these estimates fit with the results obtained in our main models. While not definitive, this provides a suggestion that mass transport supply maybe a mechanism determining the effect of closing hours on traffic accidents.

**TABLE 6:** Exploring possible mechanisms in the relationship between closing hours and traffic accidents

	Night bus cities	Least populated municipalities
	(1)	(2)
Closing hour	−6.57* (3.56)	0.109** (0.05)
R2	0.42	0.02
Observations	99	974
Municipalities	10	105

*Note*: Column (1): The dependent variable is the number of traffic accidents in the following cities: Oslo, Bergen, Stavanger, Sandnes, Trondheim, Bærum, Kristiansand, Fredrikstad, Tromsø and Drammen. Column (2): The dependent variable is the number of traffic accidents in the 25 percent least populated municipalities. All regressions are estimated with municipal fixed effects and include controls for population, number of young adults and year effects. ***, **, * indicate statistical significance at 1%, 5% and 10%, respectively.

To this point we have provided estimates of accidents at happening during a relatively broad time window. One might expect the effect of drinking hour changes to vary according to time of day, and that the effect is strongest during times when most on‐premises alcohol is consumed. We investigate this by estimating a series of regressions using different time windows. The results are presented in Table 7, where we go from a broad estimation window, including accidents happening every day but during 20:00–08:00, to a narrow definition covering accidents happening Sundays and Saturdays from midnight to 03:00.

**TABLE 7:** Differences in treatment effect by time

Days	All days	Weekend	Sat&Sun	Sat&Sun
Hours	20:00–08:00	20:00–08:00	00:00–05:00	00:00–03:00
	(1)	(2)	(3)	(4)
Closing hour	0.178* (0.094)	0.151*** (0.050)	0.133*** (0.048)	0.116*** (0.034)
Closing hour × population	−0.228* (0.130)	−0.154** (0.073)	−0.222*** (0.071)	−0.197*** (0.046)
Mean	3.59	1.15	0.548	0.132
Average % change	−1.39	−2.61	−5.48	−22.7
R2	0.313	0.251	0.168	0.132
Observations	4039	4032	4039	4039
Municipalities	423	423	423	423

*Note*: All regressions are estimated with municipal fixed effects and include controls for population, number of young adults and year effects. ***, **, * indicate statistical significance at 1%, 5% and 10%, respectively.

In the first column, we report estimates for accidents all days but only during the evening and into the morning. In an average‐populated municipality, an extension in closing hours of 1 hour will decrease all‐week accidents by 1.4%. This effect is small and statistically significant only at the 10% level. While the closing hours are also potentially binding during weekday nights, on‐premises alcohol consumption is generally lower in these times. In column (2), we further limit the estimation window to include only Fridays, Saturdays, and Sundays. We see a clear and statistically significant effect on weekend night‐time accidents, with a predicted reduction of 2.6% in accidents. In columns (3) and (4), we further narrow the estimation window to accidents after midnight Saturdays and Sundays to 05:00 and 03:00 in the morning, respectively. While the point estimates are similar to those in column (2), the underlying accident rate is smaller during these times. This leads to effect sizes of closing hour changes that are substantially larger in these time periods when hours are most likely to be binding. Most importantly, in the narrowest window, we find a decrease in accidents of almost 23%. This is, for instance, similar in magnitude to the results found in the paper by Green et al. (2014), suggesting that large changes in closing hours (such as the policy change in England and Wales) do not necessarily contribute to a larger reduction in accidents when compared to smaller liberalizations as are the typical case in our setting.

We further explore this point using an advantage of our setting insofar as we observe both liberalizations and restrictions of closing hours. This has two implications. First, it reduces the risk of unobserved variables confounding the interpretation of our estimates of closing hours. Second, it allows us to split the sample between municipalities that have liberalized and municipalities that have restricted their bar closing hours, providing the basis of a symmetry testing exercise. From a policy perspective, it is interesting to uncover whether an alteration in closing hour of a particular direction maximizes the reduction in the number of traffic accidents. There is one challenge regarding splitting up the sample in this manner. During the 10‐year period in our sample, many municipalities changed closing hours in both directions, as pointed out in Section 2. Municipalities that only liberalized or only restricted closing hours constitute around 28 and 25% of the sample, respectively. Our approach is to estimate our main model where we split the sample into municipalities that either only extended or only restricted bar closing hours.

The estimates are presented in Table 8. Column (1) reveals that the effects found in the baseline estimates are also found in municipalities that have liberalized closing hours. The coefficients of interest are larger relative to the main model, and statistically significant. Turning to column (2) reveals smaller and not statistically significant effects of closing hour restrictions on traffic accidents. These results suggest asymmetric effects of closing hours changes on traffic accidents. Reductions in opening hours have no effect on traffic accidents, but liberalization increase the number of accidents in smaller municipalities and decreases those in more populous municipalities.

**TABLE 8:** Estimated effect of opening hours on the number of traffic accidents, separated by whether hours were extended or restricted, separately (20092018)

	Only liberalized	Only restricted
	(1)	(2)
Closing hour	0.531*** (0.176)	−0.104 (0.131)
Closing hour × population	−0.501* (0.253)	0.080 (0.271)
R2	0.083	0.120
Observations	472	389
Municipalities	52	40

*Note*: The dependent variable is the number of traffic accidents occurring weekends between 10p.m. and 5a.m. All regressions are estimated with municipal fixed effects and include controls for population, number of young adults and year effects. ***, **, * indicate statistical significance at 1%, 5% and 10%, respectively.

Our data records information on the location and speed limit of the road where the accident happened. We exploit this information in columns (1) and (2) in Table 9 and differentiate between accidents occurring on urban roads where the maximum speed limit is up to, and over, 50 km per hour, respectively. The results indicate that extending closing hours increases the number of accidents on urban roads for municipalities of a smaller size. On the other hand, the average municipality experiences a reduced number of accidents in urban areas when closing hours are extended. The effects of closing hours are notably weaker for accidents on higher‐speed roads.

**TABLE 9:** Differences in treatment effect of changes in on‐premises alcohol serving hours

	Urban roads		Involved		Injuries	
	Up to 50 km/h	Over 50 km/h	One	Two or more	No or minor	Serious or fatal
	(1)	(2)	(3)	(4)	(5)	(6)
Closing hour	0.102*** (0.039)	0.035 (0.027)	0.071** (0.034)	0.066*** (0.021)	0.117*** (0.039)	0.020 (0.016)
Closing hour×	−0.124** (0.059)	−0.064* (0.036)	−0.095** (0.045)	−0.093*** (0.030)	−0.155*** (0.056)	−0.033** (0.016)
Mean	0.35	0.47	0.54	0.28	0.64	0.18
R2	0.200	0.041	0.043	0.224	0.197	0.014
Obs.	4035	4035	4035	4035	4035	4035

*Note*: The dependent variable is the number of traffic accidents occurring weekends between 10p.m. and 5a.m. All regressions are estimated with municipal fixed effects and include controls for population, number of young adults and year effects. ***, **, * indicate statistical significance at 1%, 5% and 10%, respectively.

An additional and related question is how many cars or people are involved in an accident. This is motivated by the findings of Levitt and Porter (2001) that drivers with alcohol in their blood are seven times more likely to be involved in a fatal car crash. One‐car accidents have societal costs, yet two‐car accidents involve an additional externality where drinking drivers may injure others. In our data, we can separate between single‐car accidents and accidents involving one car colliding with another car, a cyclist, or a pedestrian.^18^ The results are presented in columns (3) and (4) and suggest that changing closing hours have the same effect on accidents with one and several people involved. In terms of percentage point effects, the magnitude on both the initial effect and the interaction term are essentially the same. However, if one considers the large differences in the underlying frequency of these two events, this implies much larger effects of closing hours on several people accidents. Provided that changes in closing hour mostly affect accidents on urban roads where road user density is higher, which is the result obtained in the first two columns, it is likely that the reduction in accidents is stronger for accidents involving several people. It also suggests that extended closing hours allows patrons to disperse at more diverse times. This reduces the number of people leaving bars simultaneously with a resultant decline in accidents.

An important remaining question is how altering bar closing hours affects the severity of traffic accidents. More specifically, changing closing hours may affect the number of serious traffic accidents, which are subject to higher social costs than accidents where no one is hurt. Consequently, the treatment effect on accident severity may be of greater policy interest. Additionally, there is likely less measurement error of more serious accidents. Columns (5) and (6) in Table 9 presents the results when we split the number of accidents by the degree of severity.^19^ The effect of extending closing hours on no and minor injury accidents reflects the evidence found in the main results. Moving on to column (6), once again the direction of the coefficients mimics the results seen throughout the paper. Nevertheless, the initial effect of closing hours is not statistically significant. This result suggests that extending closing hours will lead to fewer accidents with serious or fatal injuries, irrespective of city size. Yet, the negative effect is growing with population size. Based on the mean number of accidents and the sample mean population size, liberalizing closing hours will reduce the number of accidents with minor injuries by 12%, whereas the number of serious or fatal injuries will fall by 25%. Consequently, the findings in the two latter columns imply a trade‐off for the smallest municipalities, in that increasing closing hours will increase accidents with no and minor injuries, whereas it may decrease serious and fatal accidents. The magnitude of the increase in less serious accidents is smaller, and the social cost of traffic accidents of a more serious manner is larger. In contrast, less serious accidents are more common, making it a question of assigning priorities for policymakers.

The monetary consequences associated with altering bar closing hours and the effect it has on traffic accidents can be estimated from the value of avoiding traffic accidents. This value encompasses both direct costs such as production loss, medical and material expenses, and indirect costs including pain, grief, reduced health, or reduced years of life (The Norwegian Public Roads Administration, 2018). The value of a statistical life in Norway has been calculated at $3,408,239 (in 2019 US Dollars), while the value of avoiding an accident involving a serious injury is $1,263,983. The value of preventing accidents involving a minor injury or material damages is $82,384 and $4,288, respectively. Our results indicate that extending closing hours for an averagely populated municipality will save $2990 worth of damages for accidents involving a minor injury or material damages. For serious and fatal accidents, the effects of extending closing hours will avoid accidents corresponding to a value of $93,444. In summary, a one‐hour extension aggregates to $96,434 in avoided costs per year for a municipality with the average of 12,000 inhabitants. Assuming that this effect is applicable for all Norwegian municipalities, the value of avoided accidents is $40.8 million per year.

Due to the variations by population size, we have demonstrated, these monetary effects will also be heterogeneous. For example, for municipalities with 5000 inhabitants, liberalizing closing hours will lead to an increase in less serious accidents corresponding to a cost of $1733 per year. On the other hand, liberalization will decrease serious and fatal accidents by a value of $46,722 per year. To sum up, the value of avoiding traffic accidents for less populated municipalities by changing bar closing hours appear to speak in favor for liberalization. At the same time, there are several other costs related to increased bar closing hours that is not included in this rough calculation, such as increased policing and possibly increased associated societal harms, which may alter the net welfare benefit of extended closing hours.

## 7 CONCLUSION

There is ongoing debate regarding the regulation of alcohol availability, where bar closing hours is a focus. This debate reflects a range of issues and interest groups, including perceived trade‐offs between costs associated with health and public disorder, and benefits from greater individual liberty and economic activity.

A particular focus is the link between bar closing hours and traffic safety. Existing evidence in this area, which primarily comes from one‐off extensions or restrictions, paints a mixed picture. We return to this issue focusing on Norway where municipalities are free to choose closing hours within quite large margins set nationally. Moreover, they exercise this choice, and frequently change these hours. This provides a setting where we observe many changes, both extensions and liberalizations, across a variety of time margins. Critically, this occurs in a setting where other relevant policy decisions are set nationally, and do not vary in our time of analysis.

We demonstrate average zero effects of closing hours on traffic accidents that mask large and consequential variations. A key source of variation relates to population. Later closing hours increase accidents in smaller, less populated, municipalities, while substantially decreasing accidents in average and more populated municipalities. Moreover, we also show large variations in terms of the effect of closing hours on accident severity, and the direction of the change in closing hours. Our results suggest that estimates from single policy changes may be difficult to generalize, while demonstrating that closing hours have the potential to generate large effects on traffic accidents.

Our results are important for policy purposes insofar as they demonstrate a range of potentially mediating factors influencing the effect of bar closing hours on traffic safety. A key take‐away is that the expected effect of a given closing time is highly dependent on factors such as access to public transport, population density, and differences in underlying traffic flows.

These factors should be considered closely when formulating policy in this area.

## CONFLICT OF INTEREST

We are writing to confirm that we have no conflicts of interest with respect to the publication of this paper in *Health Economics* and the research contained within. In addition, neither of the authors have received funding linked specifically to this research.

## Data Availability

The data that support the findings of this study are available from the Norwegian Institute of Public Health. Restrictions apply to the availability of these data, which were used under license for this study. Data are available from the authors with the permission of the Norwegian Institute of Public Health.

## ENDNOTES

^1^
In practice most venues increased opening times by 1–2 h.

^2^
These decisions occur at the level of 12 police districts across all of Norway, while bar hours are decided at a much more disaggregated level, across 423 municipalities.

^3^
Patrons can stay up to 30 min after alcohol sales time to finish their drinks.

^4^
The municipal structure in Norway has changed somewhat during the last decade. Municipalities that have merged are challenging to map and are therefore marked as missing.

^5^
The first quartile includes municipalities with less than 2170 inhabitants. The municipalities belonging to the second and third quartiles have between 2171 and 4500 inhabitants and 4501 and 10,000 inhabitants, respectively. The last quartile includes municipalities with more than 10,001 inhabitants.

^6^
Ridesharing has been absent in practically the whole country throughout our sample period due to strict taxi licensing laws in Norway. One exception is Uber, which was available in Oslo from 2014. However, the company operated in a very limited way and eventually withdrew from the Norwegian market in 2017.

^7^
We also estimated the regressions with standard errors clustered at the county level. This did not meaningfully change the estimated standard errors.

^8^
Oslo had approximately 674,000 inhabitants as of first of January 2018.

^9^
All models have been estimated with additional controls such as municipal average real income, education levels, unemployment rates and share of male population, which in the presence of fixed effects changes our results only negligibly.

^10^
The correlation between the two closing hours is 73 percent.

^11^
We assign the municipalities to population groups according to the population level the first year of observation, so that municipalities do not change groups over time.

^12^
We replace the zeros in the dependent variable with 0.001.

^13^
We use population levels from the first year in the sample in order not to place more weight on the latter years because of population growth.

^14^
The Norwegian word applied for closing hours is “skjenketid”, a very specific word that only refers to on‐premises alcohol serving. For traffic accident(s) or drink‐driving, we used the words “trafikkulykke”, “promillekjøring” and “fyllekjøring”.

^15^
The classification of economic regions follows the structure developed by Statistics Norway, which is based on labor and commodity market flows, and population levels.

^16^
The legal blood alcohol concentration level is 0.02 in Norway.

^17^
An incident can be reported to the police in three different circumstances. First, if a traffic accident happens, police will report the driver if they suspect that they were under the influence at the time of the accident. Second, police may stop and report a driver if the car is observed driving in a suspicious way. Third, a motorist is reported if an alcohol blood level concentration above the legal limit is revealed after roadside breath‐testing.

^18^
The last category will hereby be referred to as accidents involving several people.

^19^
In our data, minor injuries are scratches and fractures. Serious injuries include injuries that require hospital admissions, with the potential of being permanently injured.
